# LATS1 and LATS2 Phosphorylate CDC26 to Modulate Assembly of the Tetratricopeptide Repeat Subcomplex of APC/C

**DOI:** 10.1371/journal.pone.0118662

**Published:** 2015-02-27

**Authors:** Kenta Masuda, Tatsuyuki Chiyoda, Naoyuki Sugiyama, Aldo Segura-Cabrera, Yasuaki Kabe, Arisa Ueki, Koji Banno, Makoto Suematsu, Daisuke Aoki, Yasushi Ishihama, Hideyuki Saya, Shinji Kuninaka

**Affiliations:** 1 Division of Gene Regulation, Institute for Advanced Medical Research, Keio University School of Medicine, Tokyo, Japan; 2 Department of Obstetrics and Gynecology, Keio University School of Medicine, Tokyo, Japan; 3 Department of Biochemistry, Keio University School of Medicine, Tokyo, Japan; 4 Department of Molecular & Cellular BioAnalysis, Graduate School of Pharmaceutical Sciences, Kyoto University, Kyoto, Japan; 5 Division of Experimental Hematology & Cancer Biology, Cincinnati Children’s Hospital Medical Center, Cincinnati, Ohio, United States of America; 6 CREST, Japan Science and Technology Agency, Tokyo, Japan; Florida State University, UNITED STATES

## Abstract

In budding yeast, the Mitotic Exit Network (MEN) regulates anaphase promoting complex/cyclosome (APC/C) via the Dbf2-Cdc14 signaling cascade. Dbf2 kinase phosphorylates and activates Cdc14 phosphatase, which removes the inhibitory phosphorylation of the APC/C cofactor Cdh1. Although each component of the MEN was highly conserved during evolution, there is presently no evidence supporting direct phosphorylation of CDC14 by large tumor suppressor kinase 1 (LATS1), the human counterpart of Dbf2; hence, it is unclear how LATS1 regulates APC/C. Here, we demonstrate that LATS1 phosphorylates the Thr7 (T7) residue of the APC/C component CDC26 directly. Nocodazole-induced phosphorylation of T7 was reduced by knockdown of LATS1 and LATS2 in HeLa cells, indicating that both of these kinases contribute to the phosphorylation of CDC26 *in vivo*. The T7 residue of CDC26 is critical for its interaction with APC6, a tetratricopeptide repeat-containing subunit of APC/C, and mutation of this residue to Asp (T7D) reduced the interaction of CDC26 with APC6. Replacement of endogenous CDC26 in HeLa cells with exogenous phosphor-mimic T7D-mutated CDC26 increased the elution size of APC/C subunits in a gel filtration assay, implying a change in the APC/C assembly upon phosphorylation of CDC26. Furthermore, T7D-mutated CDC26 promoted the ubiquitination of polo-like kinase 1, a well-known substrate of APC/C. Overall, these results suggest that LATS1/2 are novel kinases involved in APC/C phosphorylation and indicate a direct regulatory link between LATS1/2 and APC/C.

## Introduction

The Mitotic Exit Network (MEN) in budding yeast is a signaling cascade that controls cellular events during exit from mitosis [1]. The downstream effector of the MEN is Cdc14 phosphatase, which inhibits mitotic forms of cyclin-dependent kinase (Cdk) [2,3]. The MEN also involves the Dbf2 kinase, which phosphorylates Cdc14 and causes it to relocate from the nucleolus to the cytoplasm as an active form [4]. Activated Cdc14 catalyzes the dephosphorylation of Cdh1, a cofactor of anaphase promoting complex/cyclosome (APC/C), leading to activation of APC/C^Cdh1^ and the subsequent degradation of mitotic cyclin [5,6].

Most components of the MEN are conserved from yeast to mammals. Large tumor suppressor kinase 1 (LATS1, known as the member of NDR (Nuclear Dbf2-related) kinase family) is the mammalian equivalent of the Dbf2 kinase and, like its yeast homolog, it can be activated by binding of the kinase regulator MOB1A [7–9]. Overexpression of LATS1 overcomes microtubule poison-induced mitotic arrest by facilitating mitotic exit [9], raising the possibility that LATS1 also targets CDC14 to activate APC/C. To address this hypothesis, we performed an unbiased screen of LATS1 substrates by using phosphopeptide enrichment techniques coupled with high-accuracy mass spectrometry [10]. Using this method, myosin phosphatase-targeting subunit 1 (MYPT1), but not CDC14, was identified as a substrate of LATS1. LATS1 phosphorylates and activates MYPT1, which then suppresses polo-like kinase 1 (PLK1) via dephosphorylation of its Thr210 residue [10]. The results presented here indicate that LATS1 affects not only the phosphorylation status of PLK1, but also its abundance. Given that PLK1 is a well-known substrate of APC/C^CDH1^ [11], there may be unidentified mechanisms by which LATS1 contributes to APC/C regulation in a CDC14-independent manner.

APC/C is a multi-subunit complex that functions as an E3 ubiquitin ligase during various cell cycle progressions [12]. In vertebrates, the APC/C core comprises 15 different subunits, most of which were conserved throughout evolution. Post-translational modification of APC/C components regulates the activity of the complex [13–15]. Kraft et al. [16] reported that, upon nocodazole treatment, APC/C in HeLa cell lysates is preferentially phosphorylated at its APC1 component and CDC27 (APC3), CDC16 (APC6), CDC23 (APC8), and APC7 tetratricopeptide repeat (TPR) subunits. Of the 34 mitotic *in vivo* phosphorylation sites in APC/C components, five contain the consensus motif for AGC kinase family members (R-x_1–2_-S/T) [17], suggesting the involvement of these kinases in the regulation of APC/C. LATS1 belongs to the AGC kinase family (including NDR kinase subgroup) and has a similar consensus motif to that described above [10,17]; therefore, the aim of this study was to determine whether LATS1 phosphorylates APC/C components directly. The results presented here suggest that the APC/C component CDC26 is a direct target of LATS1 and LATS2 (LATS1/2). LATS1/2 phosphorylated the N-terminal Thr7 (T7) residue of CDC26, resulting in a reduced affinity of this component for APC6, a TPR subunit of APC/C. In addition, a phosphor-mimic mutant of CDC26 (T7D) led to increased ubiquitination of PLK1 *in vivo*. These results suggest the existence of a new regulatory mechanism of APC/C that involves LATS1/2.

## Results

### Identification of CDC26 as a novel target of LATS1

To determine whether LATS1 targets APC/C components directly, APC/C was immunopurified from HeLa cells and incubated with recombinant active LATS1 (amino acids 589–1130) in an *in vitro* kinase reaction, and then the enriched phosphopeptides were subjected to high-accuracy mass spectrometry [18]. Using this method, CDC26 was identified as a potential target of LATS1 (**S1 Table**). As a positive control, a second *in vitro* kinase assay was performed using Cyclin B/CDK1; this analysis identified a number of APC/C components (**S1 Table**), including CDC27 (APC3), which have multiple mitotic Cyclin B/CDK1-specific phosphorylation sites [16], thereby confirming the reliability of the assay.

**Fig 1:**
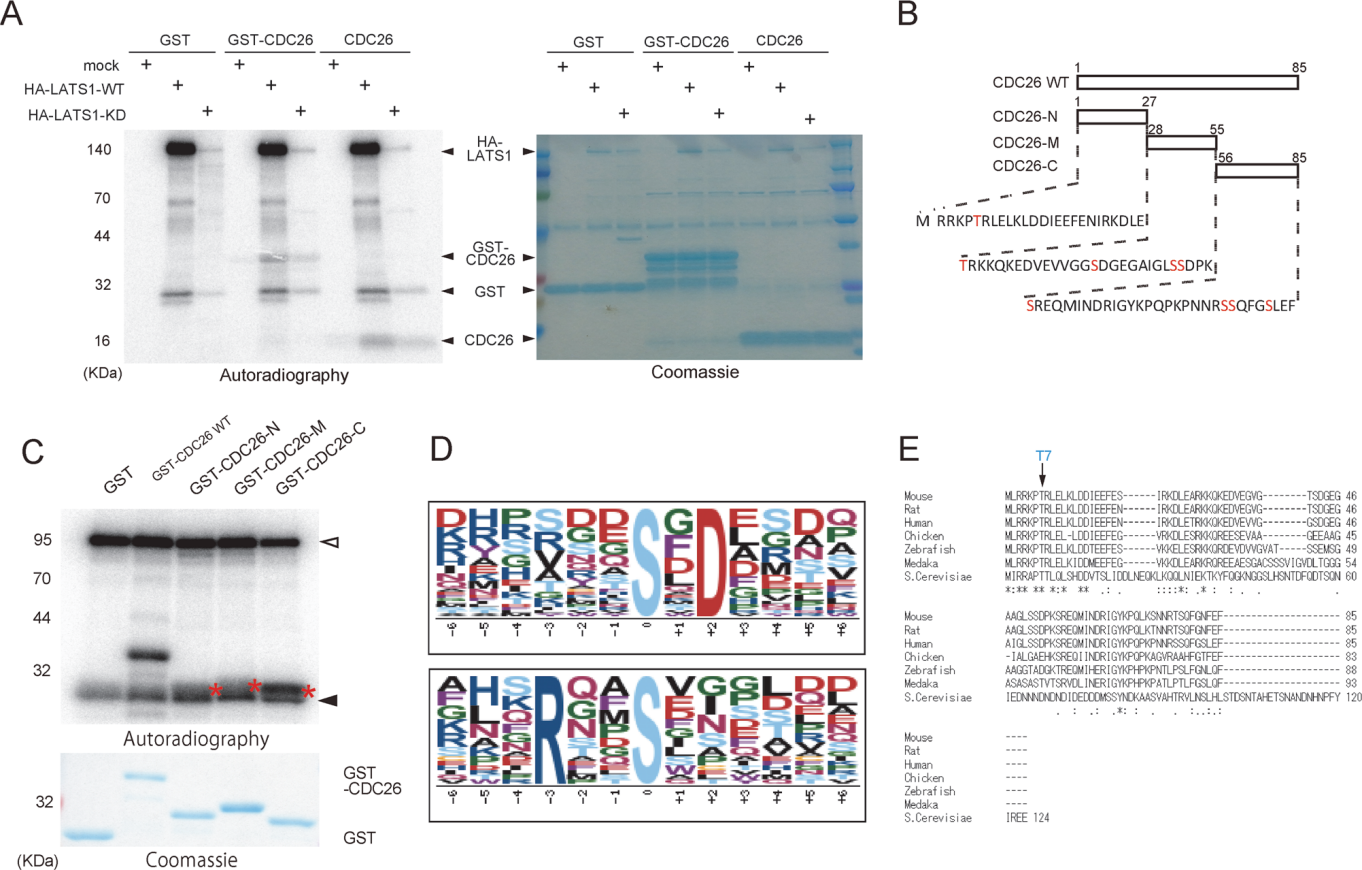
The N-terminus of CDC26 is highly conserved and contains LATS1 consensus motif. (A) Phosphorylation of CDC26 by immunopurified LATS1. HEK293T cells were transfected with a control vector or a vector expressing HA-tagged wild-type (WT) or kinase-dead (KD) LATS1, and then treated with 100 nM okadaic acid for 2 h. Lysates of the cells were immunoprecipitated with an anti-HA antibody. The immunoprecipitates were incubated with GST alone or GST-tagged CDC26 or GST-cleaved CDC26 in an *in vitro* kinase assay for 30 min at 30°C and then subjected to SDS-PAGE followed by autoradiography (left panel) and Coomassie Blue staining (right panel). (B) Schematic illustration of the human CDC26 deletion mutants and the amino acid sequences of the N-terminal region (N), C-terminal region (C), and mid-region (M) of the protein. Candidate Ser/Thr phosphorylation targets are shown in red. (C) Mapping of the region of CDC26 phosphorylated by LATS1. Active GST-LATS1 (200 ng) was incubated with GST alone or GST-tagged wild-type (WT) CDC26 or the deletion mutants shown in (B). The samples were then subjected to SDS-PAGE followed by autoradiography and Coomassie Blue staining. Asterisks depict the phosphorylated CDC26 deletion mutants. Open arrowhead indicates autophophorylated GST-LATS1. Filled arrowhead depicts non-specifically phosphorylated bands. (D) Consensus LATS1 phosphorylation motifs identified by *in vitro* kinase assays combined with phosphoproteomic approaches. (E) ClustalW alignment of the amino acid sequences of CDC26 from various species. Consensus sequences are indicated by asterisks. Double and single dots indicate the strong (>0.5) and weak (<0.5) groups of amino acids, all of which scored positively in the PAM250 matrix (Gonnet). The T7 residue matched the Arg-x-x-Ser/Thr (RxxT) LATS1 consensus motif.

**Fig 2:**
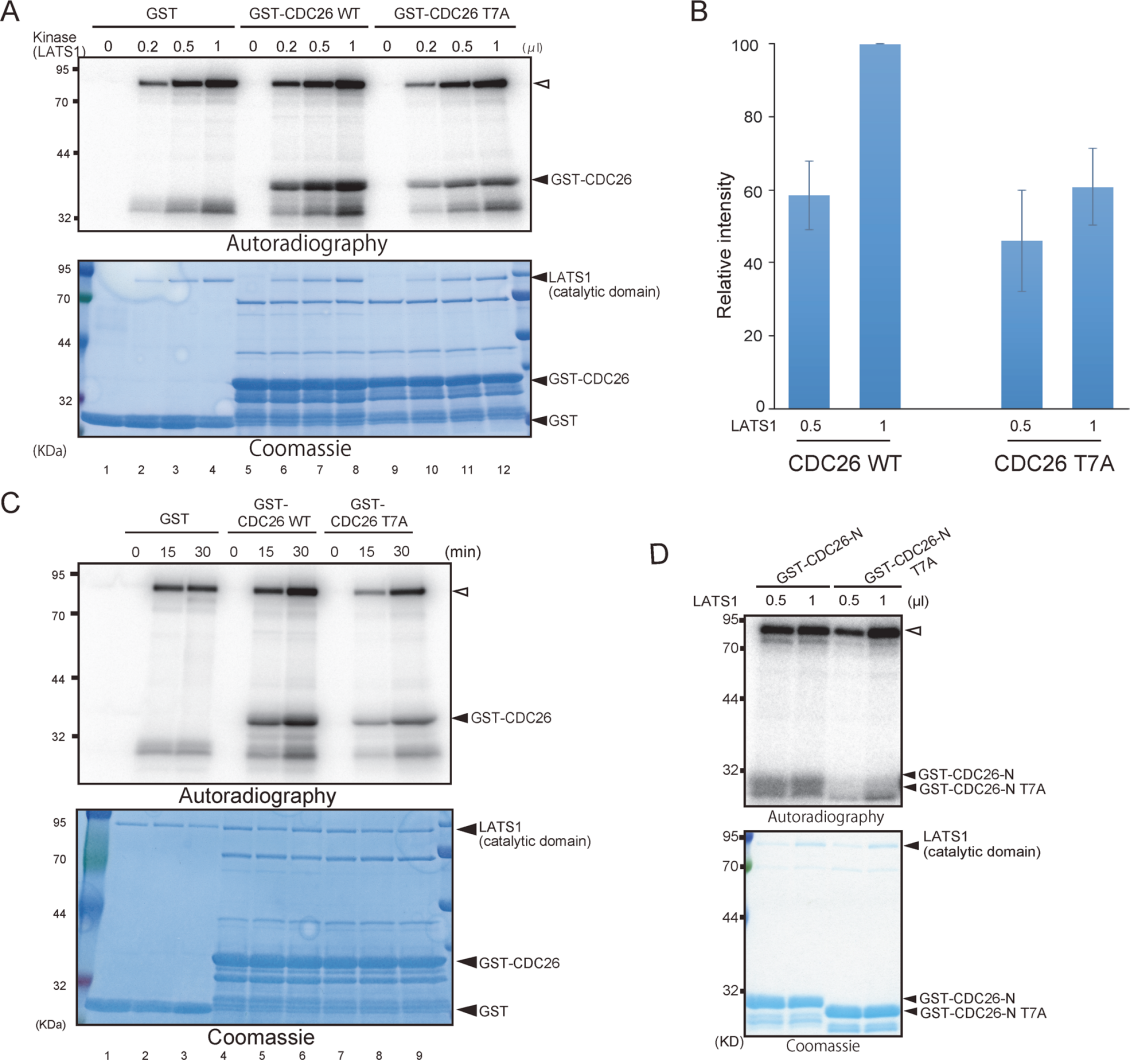
LATS1 phosphorylates the conserved N-terminal T7 residue of CDC26 *in vitro*. (A, C) Phosphorylation of CDC26 by recombinant LATS1. GST alone and GST-tagged wild-type (WT) and T7A-mutated CDC26 were incubated with the indicated amount (200ng/μl) of GST-LATS1 at 30°C for 30 min (A) or 100ng GST-LATS1 at the indicated time (C). The samples were then subjected to SDS-PAGE followed by autoradiography and Coomassie Blue staining. To adjust for differences in the reaction volume in (A), distilled water was added to the reactions in lanes 1, 5, and 9. (B) Quantitative analysis of the autoradiography data shown in (A). The intensity of each band was quantified and normalized to the intensity of the corresponding band on the Coomassie Blue-stained gel. Data are shown as the relative intensities compared with that in the reaction containing 1 μl (200 ng) of WT CDC26 and represent the mean ± standard deviation of three independent experiments. (D) GST-tagged wild-type (WT) and T7A-mutated CDC26-N (Fig. 1B) were incubated with the indicated amount (200ng/μl) of GST-LATS1 at 30°C for 30 min. The samples were then subjected to SDS-PAGE followed by autoradiography and Coomassie Blue staining. Open arrowhead indicates autophophorylated GST-LATS1.

To confirm that CDC26 is a target of LATS1, HEK293T cells were transfected with HA-tagged wild-type or kinase-dead (KD) LATS1 and then treated with okadaic acid to activate the exogenous kinase [10]. LATS1 was then immunoprecipitated using an anti-HA antibody and reacted with GST, recombinant GST-CDC26 or GST-cleaved full-length CDC26 in an *in vitro* kinase assay. Wild-type LATS1 phosphorylated CDC26 but the KD mutant did not (**Fig. 1A**). In addition, active recombinant LATS1 phosphorylated recombinant CDC26 in a dose- and time-dependent manner *in vitro* (see **Fig. 2A**, lanes 5–8; and **Fig. 2C**, lanes 4–6).

### LATS1 phosphorylates the conserved N-terminal T7 residue of CDC26

The initial screening identified the C-terminal Ser78 and Ser82 residues of CDC26 as candidate sites of phosphorylation by LATS1 (**S1 Table**). To confirm this finding, *in vitro* kinase assays were performed using deletion mutants of CDC26 (**Fig. 1B**). Consistent with the results obtained from the mass spectrometry analysis, LATS1 efficiently phosphorylated the C-terminal region of CDC26 (amino acids 56–85); however, the N-terminal region (amino acids 1–27) and mid-region (amino acids 28–55) of CDC26 were also phosphorylated by LATS1 (**Fig. 1B and C**).

His-x-Arg/His/Lys-x-x-Ser/Thr (where x denotes any amino acid) has been reported as a consensus motif for LATS1 [19]. Analysis of the immunopeptide sequences identified during the mass spectrometry screening revealed two candidate consensus motifs for LATS1, namely, Ser/Thr-x-Asp and Arg-x-x-Ser/Thr (**Fig. 1D**). We showed previously that LATS1 phosphorylates Ser445 in the Leu-Arg-Lys-Thr-Gly-Ser sequence located in the mid-region of MYPT1 [10,17]; in this sequence, the Arg residue in the LATS1 consensus motif identified here (Arg-x-x-Ser/Thr) is replaced by Lys, another basic amino acid. This region of MYPT1 is highly conserved and is important for LATS1-mediated regulation of the protein [10], highlighting the importance of the consensus sequence. Furthermore, Arg-x-x-Ser is a preferential phosphorylation site for the LATS1 homolog Dbf2 [17] [20]. Analysis of the CDC26 sequence identified only one similar consensus phosphorylation site (Arg-Lys-Pro-Thr7) at the N-terminal region. Notably, the N-terminus of CDC26 is highly conserved from yeast to human (**Fig. 1E**), and a previous structural analysis of CDC26 indicated that T7 is a key residue for the interaction with APC6 [21]. To address the significance of phosphorylation of T7 in CDC26, this residue was mutated to Ala (T7A), and an *in vitro* kinase assay was performed using wild-type or mutant full-length CDC26 and recombinant LATS1. In the presence of 1 μl (200 ng) of LATS1, mutation of T7 reduced the phosphorylation of CDC26 by approximately 60% (**Fig. 2A and B**). In addition, phosphorylation of CDC26 was attenuated by the T7A mutation in a time-dependent manner (**Fig. 2C**). When using recombinant GST-CDC26-N protein (**Fig. 1B and C**), T7A mutation also effectively eliminated the signal induced by active LATS1 (**Fig. 2D**).These results indicate that T7 of CDC26 is a LATS1 target site.

**Fig 3:**
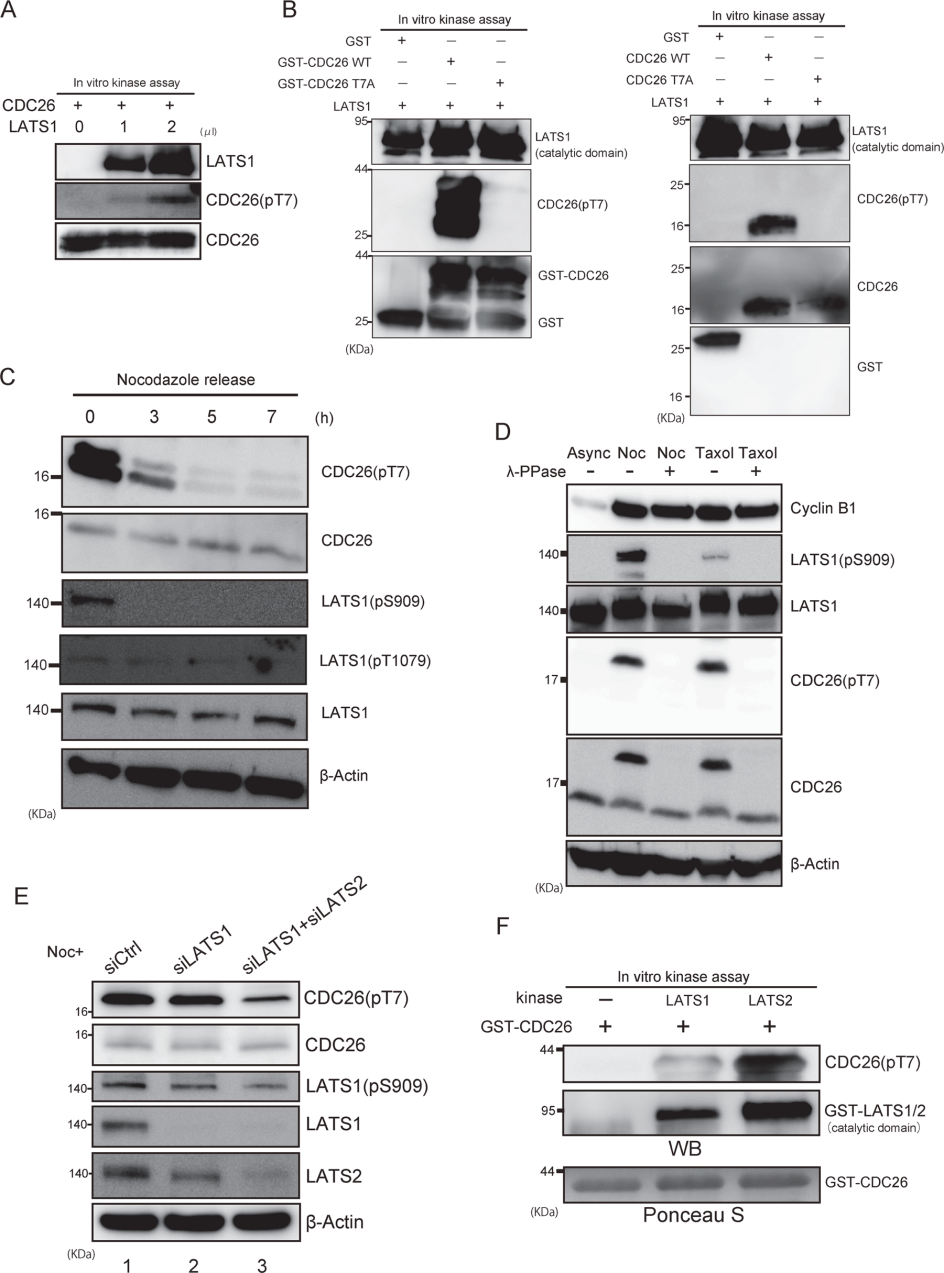
LATS1/2 phosphorylates the T7 residue of CDC26 *in vivo*. (A, B) The effects of *in vitro* incubation with LATS1 on T7-phosphorylation of CDC26. (A) Recombinant GST-tagged wild-type CDC26 was incubated with or without active GST-LATS1 and cold ATP, and then subjected to immunoblot analyses with the indicated antibodies. The control reaction (lane 1) included an equivalent amount of distilled water instead of kinase. (B) GST alone, GST-tagged recombinant wild-type (WT) or T7A-mutated CDC26 (left panel), and thrombin-cleaved WT or T7A-mutated CDC26 (right panel) were incubated with active GST-LATS1 and cold ATP for 30 min and then analyzed as described in (A). GST-CDC26 WT and other minor bands at lower molecular weight (that are possible degradation products) are observed (B, left panel). (C) Immunoblot analyses of Ser909 or Thr1079-phosphorylated LATS1 and T7-phosphorylated CDC26 levels in mitotic HeLa cells. The cells were treated with nocodazole for 16 h and then collected at the indicated times after the release from synchronization at prometaphase. (D) The effects of lambda phosphatase (λ-PPase) on the levels of Ser909-phosphorylated LATS1 and T7-phosphorylated CDC26 in extracts of HeLa cells that were grown asynchronously (Async) or treated with nocodazole (noc) or taxol for 16 h. The cell lysates were incubated with or without λ-PPase for 30 min at 30°C and then subjected to immunoblot analyses with the indicated antibodies. (E) The effects of siRNA-mediated knockdown of LATS1 and/or LATS2 on the level of T7-phosphorylated CDC26 in HeLa cells. The cells were transfected with a control, LATS1-specific, or LATS1/LATS2-specific siRNA, treated with nocodazole for 16 h to activate endogenous LATS1, and then subjected to immunoblot analyses using the indicated antibodies. (F) The effect of *in vitro* incubation with LATS2 on T7-phosphorylation of CDC26. Active GST-LATS1 and LATS2 were incubated with GST-tagged wild-type CDC26 for 30 min in the presence of cold ATP and then subjected to immunoblot analysis with the indicated antibodies. Ponceau S staining was used as a loading control.

To determine whether LATS1 phosphorylates CDC26 *in vivo*, we raised an antibody against T7-phosphorylated CDC26 (pT7). First, the specificity of the antibody was verified via immunoblot analyses of *in vitro* kinase reactions containing recombinant CDC26 with or without LATS1. As expected, the antibody signal was almost undetectable in the absence of LATS1 but increased in a dose-dependent manner in the presence of LATS1 (**Fig. 3A**). Furthermore, the pT7 antibody did not react with the T7A mutant of CDC26 (**Fig. 3B**) or CDC26-N (**S1 Fig.**), indicating a specific interaction with the phosphorylated T7 residue *in vitro*. Next, HeLa cells were treated with nocodazole to induce prometaphase arrest and activate endogenous LATS1 [22], and the phosphorylation status of CDC26 was examined by immunoblotting the cell lysates with the pT7 antibody and an antibody against total CDC26. T7-phosphorylated CDC26 was detected in prometaphase-arrested cells, and immunoblotting with antibodies against LATS1 phosphorylated at Ser909 (located in the T-loop) or Thr1079 confirmed activation of the kinase in these cells (**Fig. 3C**). LATS1 was also activated by the microtubule poison taxol (**Fig. 3D**). The band detected by the pT7 antibody, as well as the slow migrating band detected by the total CDC26 antibody after nocodazole and taxol treatment, disappeared after incubation of the cell lysates with phosphatase (**Fig. 3D**), confirming the specificity of the pT7 antibody for phosphorylated CDC26. Consistently, when CDC26 was depleted by siRNA transfection, signal of T7-phosphorylated CDC26 was decreased comparable to the level of its protein reduction (**S2 Fig.**). To confirm that LATS1 phosphorylates CDC26 *in vivo*, we examined the effect of siRNA-mediated depletion of LATS1 on nocodazole-induced phosphorylation of CDC26. Unexpectedly, phosphorylation of the T7 residue of CDC26 was still detected after knockdown of LATS1 (**Fig. 3E**, lane 2). Because Ser909 is conserved in both LATS1 and LATS2, this result was possibly a consequence of compensatory phosphorylation by LATS2 and/or other T7 kinases. In support of this prediction, double knockdown of LATS1 and LATS2 reduced the nocodazole-induced phosphorylation of T7 (**Fig. 3E**, lane 3). Consistent with this result, LATS2 also phosphorylated CDC26 efficiently both in vitro and in vivo (**Fig. 3F and S3 Fig.**). Taken together, these results indicate that CDC26 is a target of LATS1/2 *in vivo*.

### LATS1-mediated phosphorylation of the T7 residue of CDC26 inhibits the interaction between CDC26 and APC6

**Fig 4:**
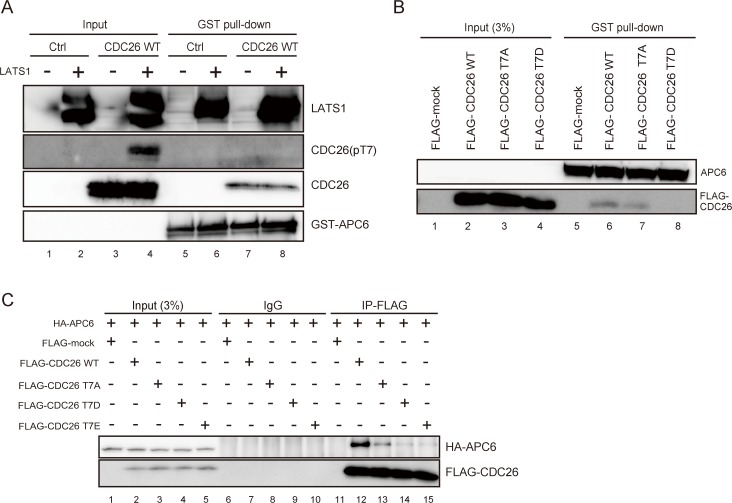
LATS1-mediated phosphorylation of CDC26 T7 inhibits the interaction between CDC26 and APC6. (A–C) Pull-down assays showing the interaction of wild-type and mutant CDC26 with APC6. (A) The interaction of T7-phosphorylated CDC26 with APC6. Recombinant CDC26 and or distilled water (Ctrl) was incubated with 0 or 2 μl (400 ng) of GST-LATS1 in a cold *in vitro* kinase assay and then incubated with GST-tagged APC6 bound to glutathione-Sepharose beads. The input fraction and the bead-bound proteins were subjected to immunoblot analyses with the indicated antibodies. (B) The interaction of the CDC26 T7A and T7D mutants with APC6. Lysates from HeLa cells expressing FLAG alone (FLAG-mock) or FLAG-tagged wild-type, T7A-mutated, or T7D-mutated CDC26 were incubated with GST-tagged APC6 bound to glutathione-Sepharose beads. The bead-bound proteins and 3% of the input fraction were subjected to immunoblot analyses using the indicated antibodies. (C) HeLa cells expressing FLAG alone (FLAG-mock) or FLAG-tagged wild-type, T7A-mutated, T7D-mutated, or T7E-mutated CDC26 were transfected with an expression vector harboring HA-tagged APC6. Lysates of the transfected cells were immunoprecipitated with control IgG and an anti-FLAG antibody. The immunoprecipitates and 3% of the input fractions were analyzed by immunoblotting with anti-HA and anti-FLAG antibodies.

Multiple proteins containing TPR motifs constitute a subcomplex of APC/C [23–26]. A rod-like structure of the N-terminal region of CDC26 is wrapped around a solenoid-like structure of eight TPRs in APC6, at which extensive hydrophobic and electrostatic interactions occur [21]. The T7 residue of CDC26 is localized proximal to, and makes a key interaction with, five residues of APC6, namely, Leu376, His406, Glu407, Val410, and Asn450 [21]; therefore, we examined whether phosphorylation of CDC26 affects its interaction with APC6. Recombinant CDC26 was incubated with cold ATP and with or without active LATS1, and then subjected to a pull-down assay using GST-tagged APC6 bound to glutathione-Sepharose beads. An immunoblot analysis using the pT7 antibody confirmed the presence of LATS1-induced T7-phosphorylated CDC26 in the input fraction (**Fig. 4A**, lane 4); however, T7-phosphorylated CDC26 was not detected in the APC6-binding fraction (**Fig. 4A**, lane 8). To rule out the possibility that the level of pulled-down CDC26 was below the limit of detection of the pT7 antibody, and to exclude the significance of other phosphorylation sites within CDC26, HeLa cells were transfected with FLAG-tagged wild-type CDC26 or a FLAG-tagged CDC26 mutant in which T7 was replaced with aspartic acid (T7D), and the cell lysates were subjected to a pull-down assay using GST-tagged APC6. Wild-type CDC26 was co-precipitated with APC6 but T7D-mutated CDC26 was not (**Fig. 4B**, lanes 6 and 8). In consistent with previous study [21], the FLAG-tagged unphosphorylated T7A mutant of CDC26 showed reduced affinity for APC6 (**Fig. 4A**, lane 7).

To analyze their interaction *in vivo*, HA-tagged APC6 was co-transfected with FLAG-tagged CDC26 into HeLa cells, and the lysates were immunoprecipitated with an anti-FLAG antibody. Consistent with the results of the *in vitro* analyses, HA-APC6 was co-precipitated with wild-type FLAG-tagged CDC26 (**Fig. 4C**, lane 12), but not phosphor-mimic mutants of CDC26 (T7D and T7E) (**Fig. 4C**, lanes 14 and 15). Furthermore, T7A-mutated CDC26 showed reduced affinity for APC6 (**Fig. 4C**, lane 13), albeit to a lesser extent than the T7D and T7E mutants.

**Fig 5:**
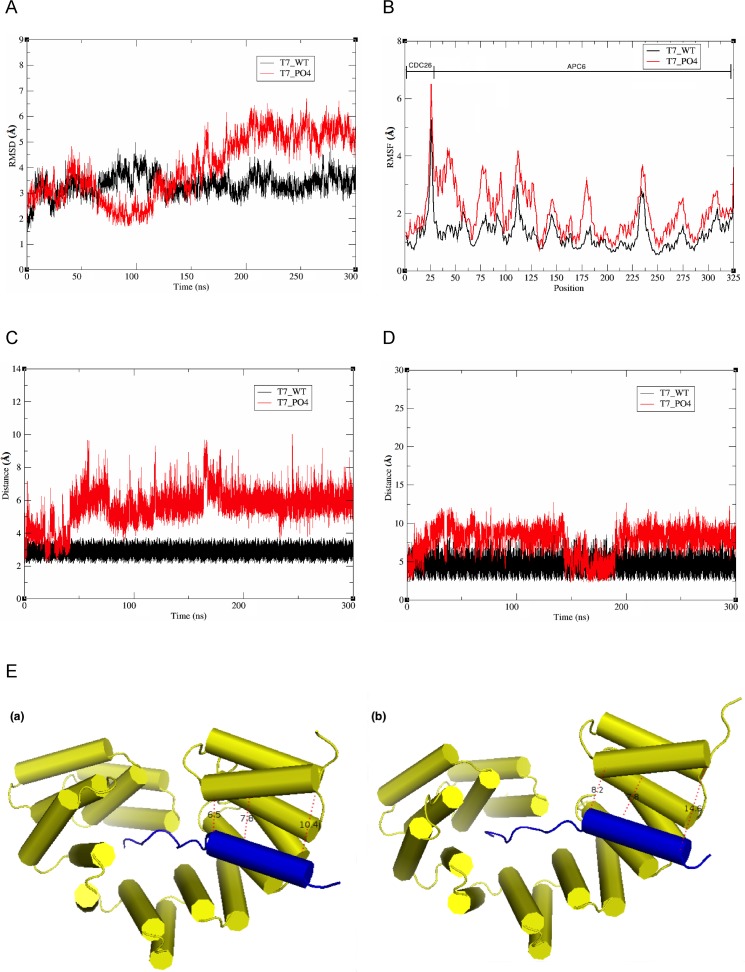
Molecular dynamics simulations of the CDC26-APC6 complex. (A) Backbone root mean square deviations (RMSDs) of the complexes comprising APC6 and T7-phosphorylated (T7_PO4) or non-phosphorylated (T7_WT) CDC26. (B) Root mean square fluctuation (RMSF) per residue for the CDC26-APC6 complexes. (C) Analysis of the interaction between T7 of CDC26 and His406 of APC6. (D) Analysis of the interaction between Phe19 of CDC26 and His521 of APC6. (E) Comparative structural analysis of interactions between helices of CDC6 (blue) and APC6 (yellow) in the complexes comprising APC6 and T7_WT (a) or T7_PO4 (b). The distances (Å) between the atoms of each amino acid are shown as dashed lines.

To further support the biochemical data showing that LATS1/2-mediated phosphorylation affects the molecular interaction between APC6 and CDC26, 300 ns molecular dynamics (MD) simulations of complexes comprising APC6 and phosphorylated (T7_PO4) or non-phosphorylated (T7_WT) CDC26 were performed. To explore the stabilities of the two complexes and the sampling method, the root mean-square deviations were determined based on the initial structures. The complex containing T7_WT reached equilibrium after approximately 100 ns, whereas the complex containing T7_PO4 showed a high degree of fluctuation for the initial 200 ns, and then stabilized thereafter (**Fig. 5A**). The root mean square fluctuation per residue was also determined to evaluate the most flexible regions of the CDC26-APC6 complex (comprising residues 1–26 from the N-terminal region of CDC26 and residues 27–325 from the TPRs of APC6, the latter of which correspond to residues 229–527 in APC6). Three distinct regions of APC6 fluctuated highly after phosphorylation of the T7 residue of CDC26, namely, residues 27–125, 170–180, and 270–280 (**Fig. 5B**). Notably, residues 27–125 (correspond to residues 229–327 in full-length APC6) constitute the site of key residues of APC6 (Tyr242, Cys307, Ile338, Ala339, His342, and Leu369) which interacts with the N-terminal region of CDC26 [21]. In addition, residues 270–280 (residues 472–482 in full-length) correspond to the non-TPR helix of APC6 that interacts with CDC26 as an intermolecular TPR mimic [21]. A long-range (300 ns) simulation of representative CDC26-APC6 interaction sites along the MD trajectories, namely, the interaction of T7 of CDC26 with His406 of APC6, and Phe19 in the CDC26 helix with His521 in the non-TPR helix of APC6, revealed a 2–4 Å dissociation of the complex containing T7_PO4 compared with that containing T7_WT (**Fig. 5C–E**). A comparison of the binding free energies of the two complexes using the MM-GBSA method supported the notion that critical interactions of the complex are affected by the phosphorylation of T7 in CDC26 (**S2 Table**). Overall, these results suggest that LATS1-mediated phosphorylation of the T7 residue of CDC26 disrupts its interaction with APC6.

### Phosphorylation of CDC26 affects APC/C assembly

**Fig 6:**
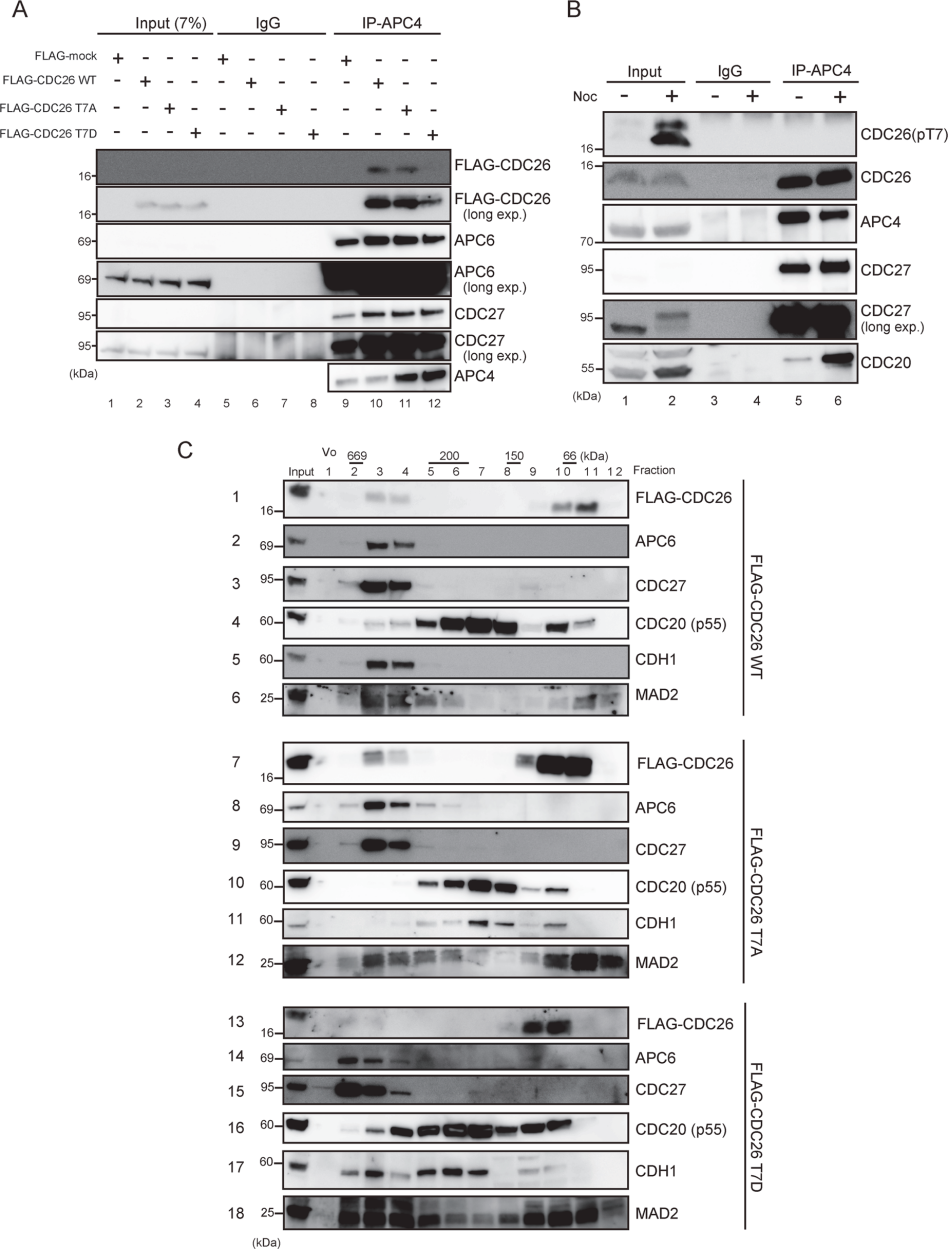
Phosphorylation of CDC26 affects APC/C assembly. (A) The effect of the T7D phosphor-mimic mutation of CDC26 on its ability to bind to APC/C. HeLa cells stably expressing FLAG alone (FLAG-mock) or FLAG-tagged wild-type, T7A-mutated, or T7D-mutated CDC26 mock were treated with a siRNA targeting the 3’UTR of endogenous CDC26. APC/C was immunoprecipitated using an anti-APC4 antibody and the immunoprecipitates, and 7% of the input fractions were analyzed by immunoblotting with the indicated antibodies. The input fractions were detected after long exposures of the blots (long exp). (B) The effect of nocodazole (noc)-induced phosphorylation of the T7 residue of CDC26 on the interaction of CDC26 with APC/C. HeLa cells were treated with or without nocodazole for 16 h and then APC/C was immunoprecipitated using anti-APC4 antibodies. The immunoprecipitates and 20% of the input fractions were analyzed by immunoblotting with the indicated antibodies. CDC27 in the input fractions were detected after long exposures of the blots (long exp). (C) Gel filtration chromatography analyses of HeLa cells stably expressing FLAG-tagged wild-type, T7A-mutated, or T7D-mutated CDC26. The cells were treated with a siRNA targeting the 3’UTR of endogenous CDC26. The lysates were fractioned on a column, and the fractions were analyzed by immunoblotting with the indicated antibodies. Vo, void volume.

To determine whether phosphorylation of T7 affects the assembly of CDC26 into APC/C, endogenous APC/C was immunoprecipitated from HeLa cells expressing FLAG-tagged wild-type or mutant CDC26 using an anti-APC4 antibody. Together with CDC27 and APC6, wild-type and T7A-mutated CDC26 were identified in the immunoprecipitates (**Fig. 6A**, lanes 10 and 11); however, T7D-mutated CDC26 was present at a much lower level (**Fig. 6A**, lane 12). Next, endogenous LATS1 activity was activated by treating HeLa cells with nocodazole [22], and then APC/C was immunoprecipitated using an anti-APC4 antibody. The total CDC26 levels were comparable in immunoprecipitates of the nocodazole-treated and untreated cells (**Fig. 6B**, row 2, lanes 5 and 6); however, T7-phosphorylated CDC26 was not co-precipitated with APC4 (**Fig. 6B**, row 1, lanes 5 and 6), suggesting that phosphorylation of the T7 residue of CDC26 is important for its disassembly within APC/C. To confirm these findings, we performed gel filtration (Superpose 6) analyses of whole-cell extracts of cells expressing FLAG-tagged wild-type, T7A-mutated, or T7D-mutated CDC26 after knockdown of endogenous CDC26. Two pools of fractions containing high and low molecular weight versions of CDC26 were generated. In addition to CDC26, the high molecular weight fractions of cells expressing wild-type or T7A-mutated CDC26 contained the TPR components APC6 and CDC27 (**Fig. 6C**, rows 1–3 and 7–9). By contrast, T7D-mutated CDC26 was significantly reduced in the high molecular weight fractions (**Fig. 6C**, row 13) supporting the notion that phosphorylation of CDC26 at T7 inhibits its interaction with APC6. In the low molecular weight fractions, CDC26 was co-eluted with CDC20 and MAD2 (**Fig. 6C**, rows 4, 6, 10, 12, 16, and 18). In addition, in the low molecular weight pool, T7D-mutated CDC26 was eluted mainly in fractions 8–10, whereas wild-type and T7A-mutated CDC26 were eluted mainly in fractions 9–11, which may reflect a conformational change of the T7D mutant caused by the phosphor-mimic amino acid substitution. Notably, the TPR components were present in fractions 2–4 of cells expressing T7D-mutated CDC26 and fractions 3–5 of cells expressing wild-type or T7A-mutated CDC26 (**Fig. 6C**, rows 14, 15 compared with rows 2, 3 and 8, 9). Furthermore, CDH1 was eluted with these APC/C components from cells expressing T7D-mutated CDC26 (**Fig. 6C**, row 17). These results suggest that the assembly of APC/C in cells containing T7-phosphorylated CDC26 differs from that in cells expressing wild-type or T7A-mutated CDC26.

### T7D-mutated CDC26 promotes the ubiquitination of PLK1 *in vivo*

**Fig 7:**
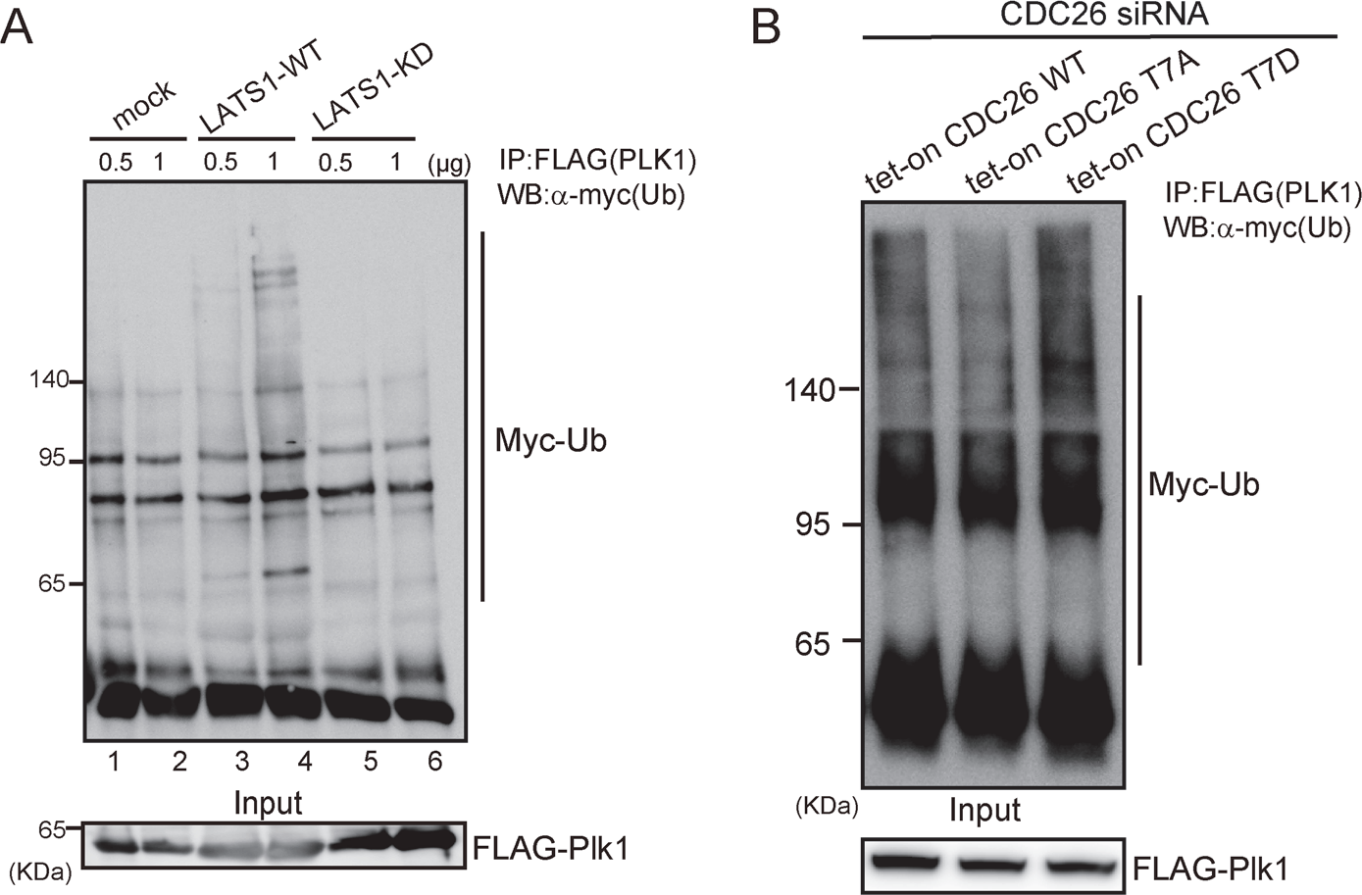
LATS1-mediated phosphorylation of CDC26 modulates the ubiquitination of PLK1 *in vivo*. (A) The effects of wild-type (WT) and kinase-dead (KD) LATS1 on the ubiquitination of PLK1 *in vivo*. HEK293T cells were co-transfected with FLAG-tagged PLK1, Myc-tagged ubiquitin and a control vector (mock) or WT or KD LATS1, and then PLK1 was immunoprecipitated using an anti-FLAG antibody. The immunoprecipitates and input fractions were analyzed by immunoblotting with antibodies against c-Myc or FLAG. (B) The effect of a phosphor-mimic mutant of CDC26 (T7D) on the ubiquitination of PLK1 *in vivo*. HeLa cells were treated with a siRNA targeting the 3’UTR of endogenous CDC26 and then induced to express exogenous wild-type, T7A-mutated, or T7D-mutated CDC26 by adding doxycycline to the culture medium. The cells were then co-transfected with FLAG-tagged PLK1 and Myc-tagged ubiquitin and treated with the proteasome inhibitor MG132 (10 μM) for 12 h prior to cell collection. PLK1 was immunoprecipitated using an anti-FLAG antibody, and the immunoprecipitates and input fractions were analyzed by immunoblotting with antibodies against c-Myc or FLAG.

Next, we examined whether LATS1-mediated phosphorylation of CDC26 affects the ubiquitination of PLK1, a known target of APC/C *in vivo*. FLAG-tagged PLK1 and Myc-tagged ubiquitin were co-expressed with wild-type or KD LATS1 in HEK293T cells, and then PLK1 was immunopurified using an anti-FLAG antibody. PLK1 was ubiquitinated in the cells expressing wild-type LATS1 in a dose-dependent manner (**Fig. 7A**, lanes 3 and 4); however, expression of KD LATS1 did not promote the ubiquitination of PLK1 (**Fig. 7A**, lanes 5 and 6). To examine the effect of the LATS1-CDC26 cascade on PLK1 ubiquitination further, HeLa cell lines expressing doxycycline-inducible wild-type, T7A-mutated, or T7D-mutated CDC26 were treated with a specific siRNA targeting the 3’ untranslated region (UTR) of endogenous CDC26 and then co-transfected with FLAG-tagged PLK1 and Myc-tagged ubiquitin. Exogenous CDC26 was induced by adding doxycycline to the culture medium. A proteasome inhibitor was added 12 h prior to cell harvesting. Ubiquitination of PLK1 was lower in cells expressing T7A-mutated CDC26 than those expressing wild-type CDC26 (**Fig. 7B**, compare lanes 1 and 2). By contrast, ubiquitination of PLK1 was upregulated in cells expressing T7D-mutated CDC26 (**Fig. 7B**, lane 3). These results suggest that LATS1-mediated phosphorylation of CDC26 at T7 has a positive effect on APC/C activity.

## Discussion

The results presented here demonstrate that CDC26 is a new substrate of the LATS1/2 kinases. Cdc26 is a non-essential heat-inducible protein in budding yeast and is required for the growth at high temperatures [27,28]. Hcn1, the fission yeast homolog of Cdc26, was identified as a high-copy suppressor of the temperature-sensitive growth phenotype of the *cut9* (*Cdc16* in budding yeast and *APC6* in vertebrates) mutant [29]. Consistent with this finding, CDC26 reportedly stabilizes the structure of APC6 [21]. CDC26 is a small protein (85 amino acids), and its N-terminal region (residues 1–24) is highly conserved from yeast to mammals (**Fig. 1E**); from a structural point of view, this conserved N-terminus is important for the ability of CDC26 to interact with APC6 [21,30]. The N-terminal region of human CDC26 forms a rod-like structure (residues 1–12) and a helix (residues 13–25). The rod-like region forms extensive hydrophobic and electrostatic interactions with the TPR domains of APC6; within this region of CDC26, T7 and Leu9 are key residues that are located proximal to five and four different residues of APC6, respectively (T7: Leu376, His406, Glu407, Val410, and Asn450; and Leu9: Phe413, Asn449, Asn450, and His453) [21]. The results presented here demonstrate that T7 is phosphorylated directly by LATS1/2 (Figs. **1–3**). *In vitro* phosphorylation of T7 or substitution of the residue with a phosphor-mimic amino acid (T7D) reduced the affinity of CDC26 for APC6 (**Fig. 4A-C**). Furthermore, MD simulations showed that the distance between T7 of CDC26 and His406 of APC6 was increased by 2-fold after phosphorylation of T7 (**Fig. 5C**). The CDC26/APC6 complex has the unique feature of an intermolecular TPR mimic parallel helix that is derived from both proteins [21]. The MD simulations also suggested that this intermolecular association was uncoupled after phosphorylation of T7 (**Fig. 5D and E**); thus phosphorylation of T7 in CDC26 appears to have a detrimental effect on the structural architecture of the CDC26/APC6 complex. In addition, the difference between the binding free energies of the CDC26/APC6 complexes containing T7-phosphorylated and unphosphorylated CDC26 was approximately 4 kcal/mol (**S2 Table**), suggesting a change in the *Kd* of approximately four orders of magnitude upon phosphorylation of T7, which is consistent with our experimental observations.

Pull-down and immunoprecipitation assays revealed that phosphorylation of T7 in CDC26 or substitution of the residue with an acidic amino acid (T7D) resulted in the release of CDC26 from APC/C (**Fig. 6A and B**). Notably, in gel filtration experiments, the TPR components of the CDC26/APC6 complex were eluted in higher molecular weight fractions from cells expressing T7D-mutated CDC26 compared with those expressing wild-type or T7A-mutated CDC26 (**Fig. 6C**). We also observed an increase in size of the TPR subcomplex in cells expressing T7D-mutated CDC26. Interestingly, expression of T7D-mutated CDC26 facilitated the ubiquitination of PLK1 *in vivo* (**Fig. 7B**). These results suggest that LATS1/2-mediated phosphorylation of CDC26 causes it to dissociate from APC/C, resulting in a positive effect on APC/C activity itself. Despite these findings, CDC26-depleted HeLa cells showed reduced cell proliferation and slower progression from prometaphase to anaphase than control cells under non-stress conditions (**S4 Fig.**), possibly reflecting attenuated APC/C activity. Therefore, a reduced amount of CDC26, which is expected to mimic CDC26 release from APC6 in a functional context, may not always promote APC/C activity. In the gel filtration assay, CDC26 was eluted in two pools of high and low molecular weight fractions (**Fig. 5C**). The low molecular weight pool also contained CDC20 and MAD2, whereas the high molecular weight pool contained these components as well as the TPR subunits. This finding raises the possibility that CDC26 regulates the activity of APC/C in a manner that is independent of its role as a constituent of the complex, which may explain the difference between the phenotypic effects of knockdown and T7D mutation of CDC26 on APC/C activity. The substrate specificity of APC/C is altered via binding of its co-activator CDC20 to different components of the complex [31]. The TPR subunits of APC/C mediate binding of the complex to the co-activator CDH1 [32]; hence, dissociation of CDC26 from the TPR subunits might change the conformation of the APC/C^CDH1^ complex to promote targeting of PLK1. Further analyses are required to elucidate the detailed molecular mechanisms and functional consequence of LATS1/2-mediated regulation of APC/C.

In addition to T7, other putative LATS1 phosphorylation sites were identified in the mid-region and C-terminal region of CDC26 (**Fig. 1B and C**). In fission yeast, Cdc26 (Hcn1) is phosphorylated at Ser48 in the mid-region of the protein, which corresponds to the consensus Cdk1 phosphorylation site [33]. However, a previous study showed that substitution of Ser48 with Asp had no effect on APC/C function; hence, the functional role of phosphorylation of this residue is somewhat uncertain.[33] Human CDC26 contains nine candidate Ser/Thr phosphorylation sites (**Fig. 1B**); however, only the T7 residue is conserved among vertebrates (**Fig. 1E**). In addition, substitution of T7 with Ala reduced LATS1-mediated phosphorylation of full-length CDC26 markedly (**Fig. 2A-C**). Along with the structural significance of T7 described above, we propose that this residue is a major phosphorylation target of the LATS1/2 kinases and plays a significant role in physiological settings. Notably, knockdown of LATS1 and LATS2 did not eliminate T7 phosphorylation completely after nocodazole treatment (**Fig. 3E**). Given that T7 is located within the consensus motif of the AGC kinase family [17], other kinases belonging to this family may also modulate the interaction between CDC26 and APC6. A previous study reported that the phosphorylation patterns of APC/C components differ in cells treated with various antimitotic drugs to induce mitotic arrest [34], suggesting the existence of some as yet unidentified APC/C kinases.

Architecture and components of MEN are conserved in fission yeast (it is called SIN, septation initiation network) [1]. However, SIN controls only the execution of cytokinesis not anaphase onset [35], indicating regulatory mechanism of mitotic exit is a complex process. Recent study of fission yeast indicates NDR kinase Sid2 (Dbf2 homologue) promotes mitotic commitment by direct phosphorylation and subsequent activation of the NIMA-related kinase Fin1 [36]. In contrast, we have reported that LATS1 suppressed G2/M transition via MYPT1-mediated suppression of PLK1 [10]. Furthermore, human CDC14 phosphatases, downstream of MEN, have been reported to have quite different functions with those of yeast Cdc14 [37]. These results imply different physiological meaning of MEN among species. Further analysis will be required to address this hypothesis.

In summary, this study demonstrates that LATS1/2 phosphorylates APC/C. The results presented here indicate that LATS1/2-mediated phosphorylation of CDC26 affects its interaction with APC6 and assembly of the TPR subunits in APC/C, leading to increased ubiquitination of PLK1 *in vivo*. These results indicate the existence of a new mechanism of regulation of APC/C in the mammalian MEN signaling pathway.

## Materials and Methods

### Cell culture, synchronization, and drug treatment

HeLa and HEK293T cells were cultured in Dulbecco's Modified Eagle's medium containing 10% fetal bovine serum and were incubated at 37°C in an atmosphere containing 5% CO_2_ [10]. HeLa cells were kind gift from Dr. Narumiya at Kyoto University and HEK293T cells were obtained from RIKEN cell bank. HeLa cells were synchronized at the G1/S boundary using a double thymidine block and release protocol (24 h incubation with 2 mM thymidine, thymidine-free incubation for 8 h, followed by incubation with 2 mM thymidine for 14 h). The cells were treated with 200 nM nocodazole (Sigma) or 50 nM taxol (Sigma) to induce prometaphase arrest. For proteasome inhibition, the cells were treated with 10 μM MG132 for 12 h prior to immunoblotting. Transient transfections were performed using FuGENE HD Transfection Reagent (Promega). Lipofectamine RNAiMAX Reagent (Invitrogen) was used to transfect cells with the RNAi oligonucleotides. The target sequences of the siRNAs used in this study were as follows: firefly luciferase (GL2 as a control), 5’-CGTACGCGGAATACTTCGAdTdT-3’; LATS1 siRNA-1, 5’-ATTCGGGAATCCCTTAGGAdTdT-3’; LATS1 siRNA-2, 5’-GCAATTGAATTCATTAGTAdTdT-3’; LATS2 siRNA, 5’-CAAGCATCCTGAGCACGCAdTdT-3’; CDC26 siRNA-1, 5’-GAGCGCTGGAATGGAATAAdTdT-3’; and CDC26 siRNA-2, 5’-TGAGATTACTGATGCTTCAdTdT-3’.

### Sample preparation and phosphoproteomic analysis

Thirty minutes after release from nocodazole-induced prometaphase arrest, HeLa cells were collected and immunoprecipitated with an anti-CDC27 antibody. *In vitro* kinase reactions were then performed using the immunoprecipitates and cold ATP. The resulting samples were analyzed by nano-liquid chromatography/tandem mass spectrometry, as described previously [10]. The LATS1 phosphorylation motifs were identified using *in vitro* kinase assays, as described previously [38]. Briefly, HeLa cell lysates were dephosphorylated with thermo-sensitive alkaline phosphatase (Promega). After heat inactivation of the phosphatase, the lysates were diluted with 40 mM Tris-HCl (pH 7.5) containing 20 mM MgCl_2_ and 1 mM ATP, and then reacted with recombinant LATS1 for 3 h at 37°C. The reaction mixture was denatured with 8 M urea followed by reductive alkylation and tryptic digestion. The phosphopeptides were enriched with hydroxyl acid-modified metal oxide chromatography [18] using titania and analyzed via nano-liquid chromatography/tandem mass spectrometry. Based on the LATS1 substrate sites identified *in vitro*, phosphorylation motifs were obtained using motif-x [39].

### Plasmid construction and retroviral gene transfer

The human *CDC26* and *APC6* coding regions were amplified by PCR using cDNA from HEK293T and HeLa cells as a template, and then subcloned into the mammalian pcDNA3 expression vector (Invitrogen). Mutations in *CDC26* were generated using the QuickChange Lightning Multi Site-Directed Mutagenesis Kit (Agilent Technologies) and confirmed by DNA sequencing. A PCR-based procedure was used to attach a HA tag to the N-terminal end of APC6. N-terminal GST-tagged versions of wild-type, T7A-mutated, and T7D-mutated CDC26 were generated by cloning the *CDC26* cDNAs into the pGEX vector. A human Gateway Entry clone encoding *PLK1* (FLJ80030AAAN) was obtained from the National Institute of Technology and Evaluation Biological Resource Center; the cDNA in this entry clone was subcloned into the destination expression vector according to the manufacturer’s instructions (Invitrogen). pcDNA3 FLAG-LATS2 was kindly provided by Dr. Yamamoto at Tokyo University. A 3× FLAG tag was added to the C-terminal end of wild-type, T7D-mutated, and T7A-mutated CDC26 by cloning the *CDC26* cDNAs into the pMXs-3×FLAG-IP vector. The pMXs vectors were then transfected into Plat-A packaging cells, and transfectants were selected by culturing in the presence of 2 μg/ml puromycin for 7 days. Myc-tagged ubiquitin was generated by subcloning the cDNA encoding human ubiquitin into the pBJ-Myc vector. The custom pRetroX-Tet-On Advanced-hygro vector was constructed by digesting pRetroX-Tet-On Advanced with BstXI and XhoI to remove the neomycin- and kanamycin-resistance cassette, which was then replaced with a hygromycin resistance gene. The pRetroX-Tight-Pur-CDC26 vector was constructed by cloning CDC26 into pRetroX-Tight-Pur at the BamHI and NotI sites. Double stable HeLa cells expressing RetroX-Tet-On Advanced-hygro and pRetroX-Tight-Pur-CDC26 were generated by serial infection, according to the manufacturer’s instructions (Clonetech).

### Expression and purification of GST fusion proteins

GST fusion proteins expressed in *Escherichia coli* BL21 cells (BioDynamics Laboratory) were extracted with BugBuster Protein Extraction Reagent (Novagen) and isolated using glutathione-Sepharose beads (GE Healthcare). The GST fusion proteins were eluted from the beads by incubating with 15 mM glutathione and were dialyzed prior to use. To remove the GST tag, the GST fusion proteins were incubated with 80 U/ml thrombin (GE Healthcare) for 4 h at 22°C. Thrombin was removed using Benzamidine Sepharose 6B, according to the manufacturer’s instructions (GE Healthcare).

### Immunoprecipitation, GST pull-down, protein dephosphorylation, immunoblotting, and antibodies

For immunoprecipitation and GST pull-down assays, transfected cells were washed with phosphate-buffered saline (PBS) and solubilized with lysis buffer [0.5% NP-40, 25 mM Tris-HCl (pH 7.5), 137 mM NaCl, 1 mM EDTA, 5% glycerol, 1 mM dithiothreitol (DTT), 20 mM β-glycerophosphate, 1 mM sodium vanadate, and protease inhibitor (Roche)]. The cell lysates were centrifuged at 14,000 g for 20 min at 4°C and the supernatants were incubated for 1 h at 4°C with specific antibodies immobilized on magnetic Dynabeads (Invitrogen), or GST fusion proteins immobilized on glutathione-Sepharose beads (GE Healthcare). The beads were then separated using a magnetic field or centrifugation and washed with lysis buffer. The bound proteins were eluted by boiling for 5 min in sodium dodecyl sulfate (SDS) sample buffer and then subjected to immunoblotting. For protein dephosphorylation, the cell lysates were treated with 800 units of λPPase (New England BioLabs Inc.) and incubated at 30°C for 30 min. The reaction was terminated by adding SDS sample buffer and boiling for 5 min. For immunoblotting, proteins were separated via SDS-polyacrylamide gel electrophoresis (SDS-PAGE) and then transferred electrophoretically to a polyvinylidene difluoride membrane (Millipore). The membranes were blocked with 5% non-fat dried milk in PBS for 30 min at room temperature and then exposed to the primary antibodies for 24 h at 4°C. The following mouse monoclonal antibodies were used: anti-c-Myc (9E10) (1:500 dilution; sc-40, Santa Cruz Biotechnology), anti-FLAG M2 (1:1000 dilution; F1804, Sigma-Aldrich), anti-Cdc27(AF3.1) (1:1000 dilution; sc-9972, Santa Cruz Biotechnology), anti-β-actin (AC-15) (1:2000 dilution; sc-69879, Santa Cruz Biotechnology), anti-cyclin A (1:500 dilution; 611268, BD Biosciences), anti-cyclin B1 (GNS1) (1:1000 dilution; sc-245, Santa Cruz Biotechnology), anti-GST (1:1000 dilution; GS019, Nacalai Tesque), anti-P55 Cdc (H-7) (1:500 dilution; sc-5296, Santa Cruz Biotechnology), and anti-Cdh1 (DH01) (1:500 dilution; CC43, Calbiochem). The rabbit monoclonal anti-APC6 (D8D8) (1:1000 dilution; #9499, Cell Signaling Technology) antibody was used. The following rabbit polyclonal antibodies were used: anti-APC4 (1:500 dilution; ab72149, Abcam), anti-PLK1 (1:500 dilution; A300-251A, Bethyl Laboratories Inc.), anti-LATS1 (1:1000 dilution; A300-478A, Bethyl Laboratories Inc.), anti-LATS2 (1:500 dilution; A300-479A, Bethyl Laboratories Inc.), anti-UbcH10 (1:500 dilution; A-650, BostonBiochem), anti-Cdc26/Apc12 (1:250 dilution; ab96346, Abcam), anti-Phosho-LATS1 (Ser909, Thr1079) (1:500 dilution; #9157, #8654, Cell Signaling Technology), anti-Claspin (1:500 dilution; A300-267A, Bethyl Laboratories Inc.), and anti-PTTG-1 (1:500 dilution; 34–1500, Invitrogen). The rat monoclonal anti-HA (1:2000 dilution; 3F10, Roche) and the goat polyclonal anti-APC4 (C-18) (1:500 dilution; sc-21414, Santa Cruz Biotechnology) antibodies were used. After incubation with the primary antibodies, the membranes were washed three times with PBS containing 0.1% Tween 20 and then incubated with HRP-conjugated secondary antibodies (1:2000 dilution; GE Healthcare) for 40 min. After three washes with PBS containing 0.1% Tween 20, the proteins were detected using ECL Plus Western Blotting Detection Reagent (GE Healthcare) and a chemiluminescence detector (LAS-3000; Fujifilm). Rabbit antiserum against Mad2 was generated as described previously [40]. A rabbit polyclonal antibody specific for T7-phosphorylated CDC26 was generated by affinity purification and removal of non-phospho antibodies from whole rabbit serum that was prepared by repeated immunizations with an epitope tag peptide (LRRKPpTRLELC). A cysteine residue was added to the C-terminal end of the epitope tag peptide to facilitate coupling to keyhole limpet hemocyanin.

### *In vitro* kinase assay

GST-LATS1 (amino acids 589–1130) and GST-LATS2 (amino acids 553–1088) were obtained from Carna Biosciences Inc. The 20 μl kinase reactions [20 mM Tris (pH 7.4), 5 mM MgCl_2_, 1.8 μM cold ATP, 18 μCi of γ-[32P] ATP (6,000 Ci/mmol; Institute of Isotopes Co. Ltd.), and 1 mM DTT] were incubated at 30°C for 30 min. The reactions were terminated by the addition of 4× SDS sample buffer and then boiled at 95°C for 5 min. The samples were separated by SDS-PAGE (5–20% gradient gels), visualized by Coomassie Blue staining, and subjected to autoradiography. The intensities of the bands were quantified using a BAS-5000 phosphoimager (Fujifilm). For cold *in vitro* kinase assays, the 20 μl reactions [20 mM Tris (pH 7.4), 5 mM MgCl_2_, 1 mM cold ATP, and 1 mM DTT] were incubated at 30°C for 30 min, terminated by the addition of 4× SDS sample buffer, and then boiled at 95°C for 5 min. The samples were separated by SDS-PAGE (5–20% gradient gels) and subjected to immunoblot analyses.

### *In vivo* ubiquitination assay

Twenty-four hours after transfection with plasmids encoding Myc-tagged human ubiquitin or FLAG-tagged PLK1, HEK293T and HeLa cells were incubated with 10 μM MG132 for 12 h before harvest. The cells were then collected, immunoprecipitated using an anti-FLAG antibody, and immunoblotted using an anti-Myc antibody to detect polyubiquitination.

### Gel filtration

The formation of APC/C was examined via gel filtration chromatography using the SMART system and a Superose 6 PC 3.2/30 column (GE Healthcare). HeLa cells expressing FLAG-tagged wild-type, T7A-mutated, or T7D-mutated Cdc26 were treated with a siRNA against endogenous CDC26 and seeded into 10 cm dishes. The cells were collected, washed with PBS, and lysed in 150 μl of lysis buffer. The lysates were then centrifuged at 15,000 g for 15 min at 4°C, and the supernatants were filtered with a 0.22 μm filter unit (Millipore). The samples were loaded onto a Superose 6 PC 3.2/30 column that had been equilibrated with 0.5% NP-40 lysis buffer. The column was run at a flow rate of 40 μl/min in buffer comprising 150 mM NaCl, 50 mM HEPES-KOH (pH 7.5), and 0.1 mM DTT. Aliquots of the fractions (100 μl) were analyzed by immunoblotting. Protein sizes were estimated using molecular mass size markers (thyroglobin, 669 kDa; catalase, 232 kDa; aldolase, 150 kDa; bovine albumin, 66 kDa; and β-amylase, 20 kDa).

### Time-lapse imaging

HeLa cells stably expressing GFP-tagged histone H2B were seeded onto a glass-bottomed dish (Iwaki). Time-lapse fluorescence microscopy and differential interference contrast video microscopy were performed using an Olympus LCV110 microscope. Images were acquired using a charge-coupled device camera (Retiga EXi, QImaging) equipped with a U Plan Super Apochromatic ×40, 0.95 numerical aperture objective, and then analyzed using MetaMorph software (Molecular Devices).

### MD simulations

The initial coordinates of the CDC6-APC6 complex [21] were obtained from the Protein Data Bank (PDB ID: 3HYM). MD simulations of complexes containing unphosphorylated and T7-phosphorylated CDC26 were performed using the AMBER 12.0 package and ff12SB force field [41]. The force field parameters for phosphor-Thr were obtained from the AMBER parameter database [42]. To ensure overall electroneutrality of the system, appropriate numbers of counter ions (Na^+^) were added to the most electronegative areas of the protein. The entire protein was subsequently solvated in a box of TIP3P water molecules [43] with a margin of 12.0 Å from the solute in each direction. The particle mesh Ewald method was used to calculate long-range electrostatic interactions. The cutoff distance for the long-range electrostatic and van der Waals energy terms was set at 8.0 Å. The SHAKE algorithm was used to constrain all covalent bonds involving hydrogen atoms [44], allowing for an integration time step of 2 fs. To avoid edge effects, the periodic boundary conditions were applied in the MD simulations [44]. For equilibration, the solvent molecules and counter ions, the side chains of the protein, and the whole systems were successively minimized using the steepest descent method (5000 steps), followed by the conjugated gradient method (5000 steps), while the remainder of the protein was restrained using a force constant of 500, 10, and 0 kcal/mol-Å^2^, respectively. Each system was then heated gradually from 0 to 300 K over a period of 50 ps and maintained at 300 K with a coupling coefficient of 1.0/ps and a force constant of 1.0 kcal/mol-Å^2^ on the complex. Each system was equilibrated to a free simulation for 1000 ps. Finally, a production run was performed for 300 ns using the NPT ensemble at 300 K with 1.0 atm pressure. During the MD simulation process, coordinates were collected every 1 ps. For each system, free energy calculations were performed for 4000 snapshots extracted from the last 40 ns of the stable MD trajectory using the MM-GSA approach. For each snapshot, the free energy was calculated for each molecular species (complex, protein, and ligand). The MMGBSA method can be conceptually summarized as follows:

 ΔGbind = Gcomplex—(Gprotein + Gligand) (1)

The Δ*G*_*bind*_ was determined as follows:

The binding free energy (Δ*G*_*bind*_) is evaluated a sum of the changes in the molecular mechanical gas-phase binding energy (Δ*G*_MM_), the solvation free energy (Δ*G*_solv_), and entropic (-*T*Δ*S*) contribution:

 ΔGbind = ΔGMM + ΔGsolv—TΔS (2)

The molecular mechanics energy (G_*MM*_) comprised the electrostatic (*G*_*ele*_) and van der Waals (*G*_*vdw*_) interactions:

 ΔGMM = ΔGele + ΔGvdw (3)

The solvation free energy (G_*solv*_) comprised polar (*G*_*pol*_) and nonpolar (*G*_*nonpol*_) contributions:

 ΔGsolv = ΔGpol + ΔGnonpol (4)

The Δ*G*_*pol*_ value was obtained using the PBSA module of the AMBER package. The Δ*G*_*nonpol*_ term was determined as follows, where γ (the surface tension) and *b* (a constant) were set to 0.0072 kcal/mol-Å^2^ and 0, respectively, and SASA was the solvent accessible surface area (Å^2^) determined using the linear combination of the pairwise overlaps model:

 ΔGnonpol = γSASA + b (5)

The conformational entropic contribution (*TΔS*) was estimated for 50 snapshots taken from the last 40 ns of stable simulation using the mmpbsa_py_nabnmode module implemented in Amber Tools 14. The comparative structural analysis was performed by using a representative snapshot from the clustering of the trajectories of the last 20 ns of stable MD simulation.

### Statistical analysis

Statistical analyses were performed using two-tailed Student’s *t*-tests. *P* < 0.05 was considered significant.

## Supporting Information

S1 Table Phosphoproteomic screening of in vitro LATS1 kinase reaction of immunopurified APC/C. Screening was performed as described in the “Materials and Methods”. Modified residue of each peptide and their protein description were indicated.
(XLSX)

S2 Table Binding free-energy calculation for each APC/C complex containing WT or T7-phosphorylated (T7_PO4) CDC26. (XLSX)

S1 Fig LATS1/2 phosphorylates the T7 residue of CDC26-N in vitro. GST alone, GST-tagged recombinant wild-type (WT) or T7A-mutated CDC26 were incubated with active GST-LATS1 (A) or GAT-LATS2 (B) and cold ATP, and then subjected to immunoblot analyses with the indicated antibodies.
(TIF)

S2 Fig Immunoblot analyses of T7-phosphorylated CDC26. HeLa cells were transfected with a control or CDC26-specific siRNA, treated with nocodazole for 16 h to activate endogenous LATS1, and then subjected to immunoblot analyses using the indicated antibodies.
(TIF)

S3 Fig LATS2 overexpression resulted in the increased T7 phosphorylation of CDC26. HeLa cells were transfected with a control or LATS2 expression vector, treated with nocodazole, and then subjected to immunoblot analyses using the indicated antibodies.
(TIF)

S4 Fig Cell proliferation and mitotic progression after knockdown of CDC26 or LATS1/2. (A) The effects of siRNA-mediated knockdown of CDC26 on the proliferation of HeLa cells. The cells were transfected with a control or two different CDC26-specific siRNAs, and cell viability assays were performed on days 0–3 after transfection. (B) The effects of knockdown of CDC26 on cell cycle progression. HeLa cells stably expressing GFP-tagged histone H2B were transfected with a control or CDC26-specific siRNA and synchronized at the G1/S phase by double thymidine block. The time at which nuclear envelope breakdown occurred was set as 0 min and the percentages of cells entering anaphase were counted in three different fields of time-lapse microscopy images. Data are represented as the mean ± standard deviation of these counts. (C) HeLa cells were transfected with a control or LATS1/2 siRNA oligos and cell viability was assessed as in (A). (D) siRNA-mediated knockdown of LATS1 caused the mitotic exit delay. HeLa cells were transfected with a control or two different LATS1-specific siRNAs and treated with nocodazole to arrest at prometaphase. After washing out the nocodazole, percentage of mitotic cells was evaluated by phosphor-Histone H3 and propidium iodide staining. Percentage of cells at mitosis (square region, left) was counted at the indicated time after release (right). (*, P < 0.05, Student’s two-tailed *t* test).
(TIF)
