# Correction: LATS1 and LATS2 Phosphorylate CDC26 to Modulate Assembly of the Tetratricopeptide Repeat Subcomplex of APC/C

**DOI:** 10.1371/journal.pone.0125308

**Published:** 2015-04-10

**Authors:** Kenta Masuda, Tatsuyuki Chiyoda, Naoyuki Sugiyama, Aldo Segura-Cabrera, Yasuaki Kabe, Arisa Ueki, Koji Banno, Makoto Suematsu, Daisuke Aoki, Yasushi Ishihama, Hideyuki Saya, Shinji Kuninaka

The seventh author’s name is spelled incorrectly. The correct name is: Kouji Banno.
