# Supplementary figures and images for: LATS1 and LATS2 Phosphorylate CDC26 to Modulate Assembly of the Tetratricopeptide Repeat Subcomplex of APC/C
Source: PLoS One. 2015 Feb 27;10(2):e0118662. doi: 10.1371/journal.pone.0118662 (PMC4344199; doi:10.1371/journal.pone.0118662)

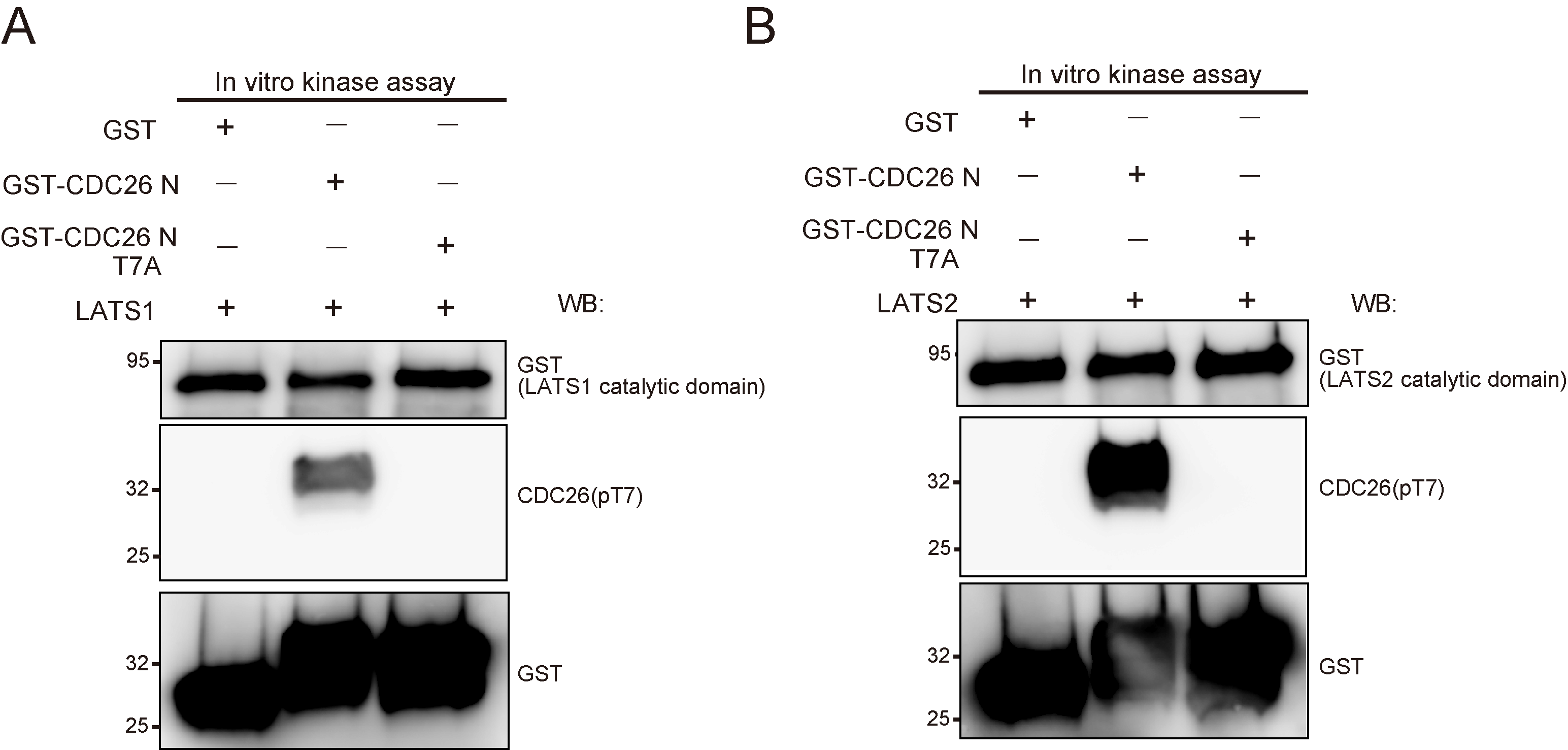

Supplement: S1 Fig — GST alone, GST-tagged recombinant wild-type (WT) or T7A-mutated CDC26 were incubated with active GST-LATS1 (A) or GAT-LATS2 (B) and cold ATP, and then subjected to immunoblot analyses with the indicated antibodies. (TIF) [file pone.0118662.s003.tif]

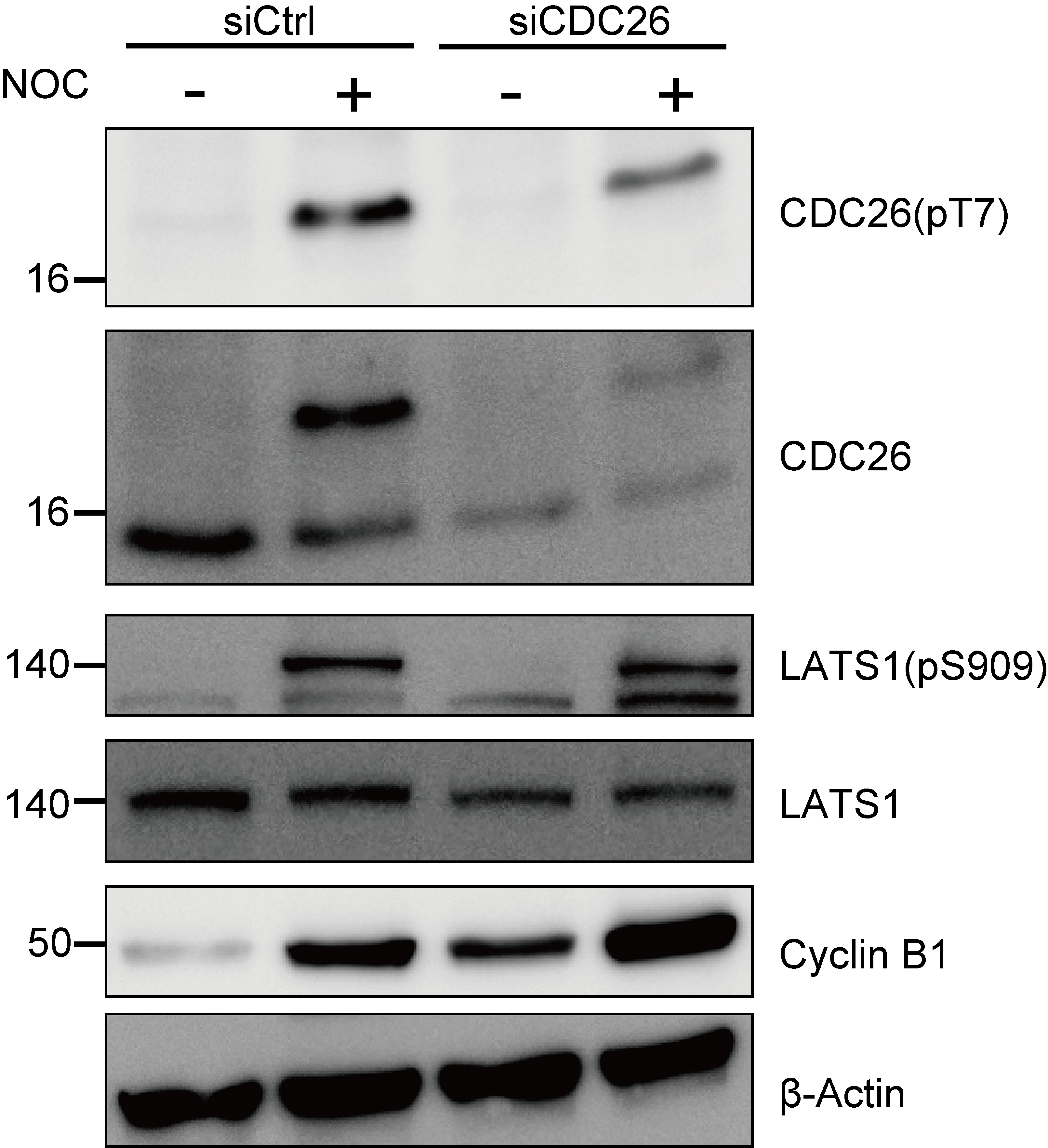

Supplement: S2 Fig — HeLa cells were transfected with a control or CDC26-specific siRNA, treated with nocodazole for 16 h to activate endogenous LATS1, and then subjected to immunoblot analyses using the indicated antibodies. (TIF) [file pone.0118662.s004.tif]

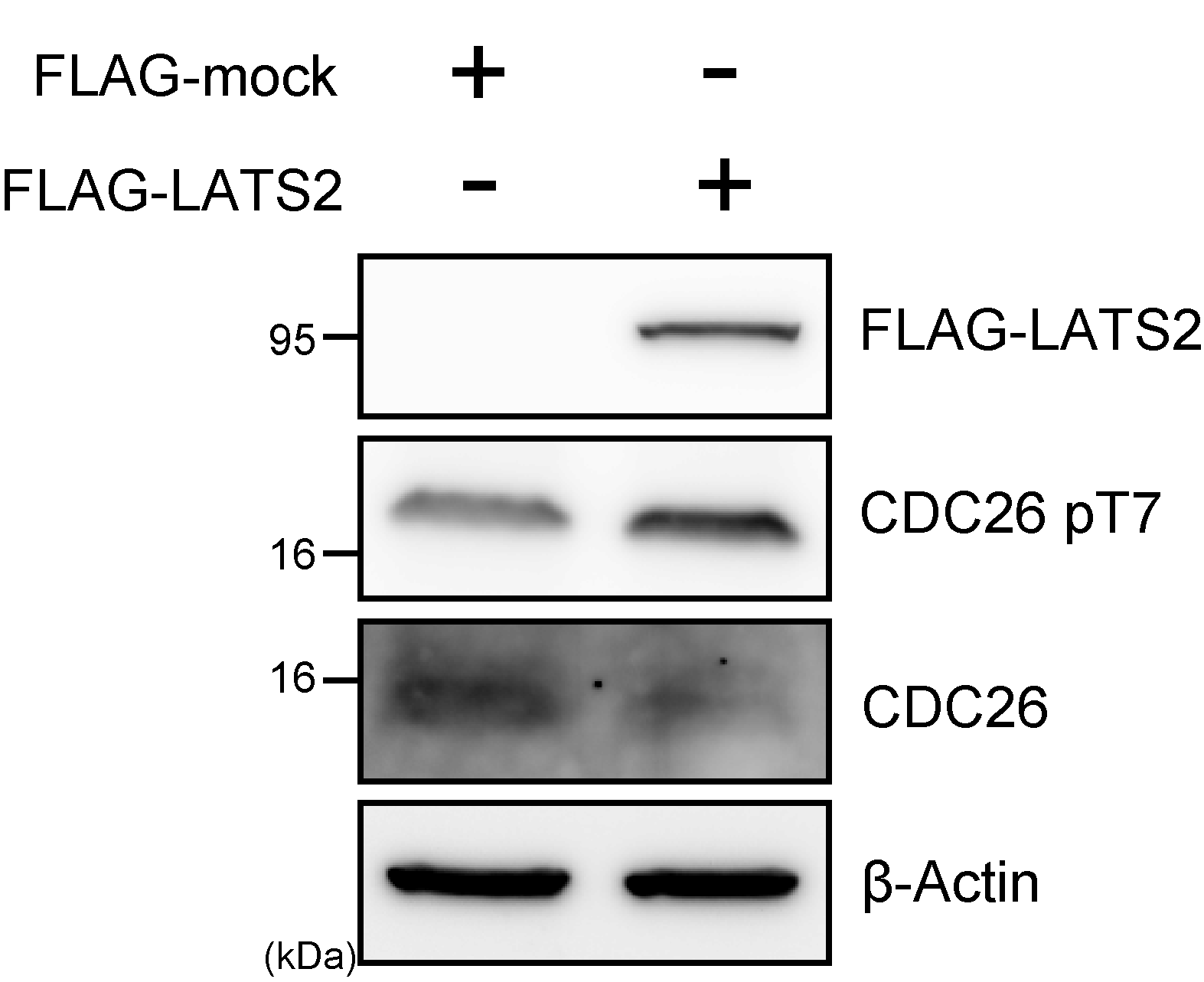

Supplement: S3 Fig — HeLa cells were transfected with a control or LATS2 expression vector, treated with nocodazole, and then subjected to immunoblot analyses using the indicated antibodies. (TIF) [file pone.0118662.s005.tif]

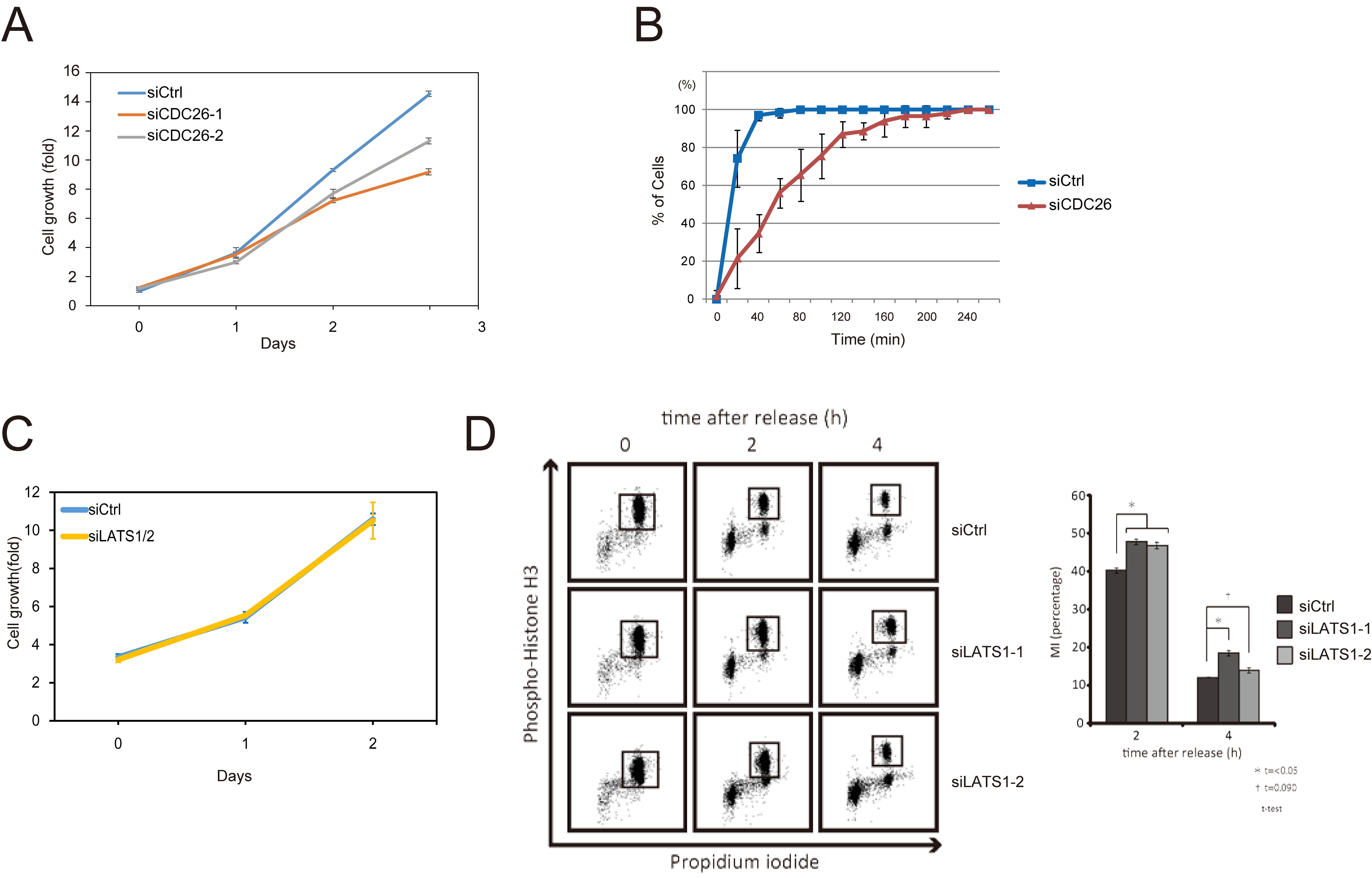

Supplement: S4 Fig — (A) The effects of siRNA-mediated knockdown of CDC26 on the proliferation of HeLa cells. The cells were transfected with a control or two different CDC26-specific siRNAs, and cell viability assays were performed on days 0–3 after transfection. (B) The effects of knockdown of CDC26 on cell cycle progression. HeLa cells stably expressing GFP-tagged histone H2B were transfected with a control or CDC26-specific siRNA and synchronized at the G1/S phase by double thymidine block. The time at which nuclear envelope breakdown occurred was set as 0 min and the percentages of cells entering anaphase were counted in three different fields of time-lapse microscopy images. Data are represented as the mean ± standard deviation of these counts. (C) HeLa cells were transfected with a control or LATS1/2 siRNA oligos and cell viability was assessed as in (A). (D) siRNA-mediated knockdown of LATS1 caused the mitotic exit delay. HeLa cells were transfected with a control or two different LATS1-specific siRNAs and treated with nocodazole to arrest at prometaphase. After washing out the nocodazole, percentage of mitotic cells was evaluated by phosphor-Histone H3 and propidium iodide staining. Percentage of cells at mitosis (square region, left) was counted at the indicated time after release (right). (*, P < 0.05, Student’s two-tailed t test). (TIF) [file pone.0118662.s006.tif]
